# Mechanical Thrombectomy for Anterior Cerebral Artery Occlusion in a Patient With a Persistent Trigeminal Artery: A Rare Anomaly With Complex Comorbidities

**DOI:** 10.7759/cureus.83160

**Published:** 2025-04-28

**Authors:** Maitha K Alnuaimi

**Affiliations:** 1 Internal Medicine, Tawam Hospital, Abu Dhabi, ARE

**Keywords:** anterior cerebral artery occlusion, chronic kidney disease, mechanical thrombectomy, persistent trigeminal artery, stroke

## Abstract

Anterior cerebral artery occlusion is a critical cause of ischemic stroke with potentially severe outcomes if not treated promptly. Mechanical thrombectomy has become the cornerstone therapy for large vessel occlusions in the anterior circulation. However, the presence of a persistent trigeminal artery, a rare embryological anastomosis, can introduce diagnostic and technical complexities, especially in patients with significant comorbidities such as hypertension, diabetes, heart failure, and chronic kidney disease.

We describe a 56-year-old woman with poorly controlled diabetes mellitus, severe hypertension, hyperlipidemia, advanced stage five chronic kidney disease, and coronary artery disease who was initially found unconscious. Upon regaining consciousness, she presented with dense left-sided hemiparesis, facial weakness, dysarthria, and eye deviation, resulting in a National Institutes of Health Stroke Scale score of 12. A non-contrast head computed tomography scan revealed hypodensity in the right cerebellum; however, subsequent computed tomography angiography confirmed an occlusion of the anterior cerebral artery, consistent with an anterior circulation stroke. Digital subtraction angiography further revealed a persistent trigeminal artery originating from the right internal carotid artery and contributing to the supply of the anterior cerebral artery. Urgent mechanical thrombectomy led to successful recanalization. Despite this, her neurological recovery remained limited, likely due to advanced atherosclerosis, multiple comorbidities, and some delay in treatment. She required prolonged rehabilitation and assistance with activities of daily living.

This case emphasizes the need for rapid assessment and endovascular intervention in anterior cerebral artery occlusions, even when complicated by unusual vascular variants and severe systemic disease. A persistent trigeminal artery can alter expected hemodynamic patterns and pose technical challenges during thrombectomy. Multidisciplinary collaboration, individualized care, and meticulous risk-factor management are pivotal to optimizing outcomes in these complex scenarios.

## Introduction

Anterior cerebral artery occlusion (ACAO), akin to other anterior circulation large-vessel occlusions, can be a devastating cause of ischemic stroke. Prompt restoration of blood flow is crucial to reduce the high rates of mortality and morbidity. Multiple large randomized trials have firmly established mechanical thrombectomy as a mainstay of therapy for acute large vessel occlusions in the anterior circulation [1,2].

However, a persistent trigeminal artery (PTA) introduces additional complexity. A PTA is a fetal anastomosis linking the internal carotid artery (ICA) with arterial segments that can variably contribute to either the vertebrobasilar system or certain anterior circulation variants [3]. Although exceedingly rare, the PTA may affect collateral routes, diagnostic clarity, and endovascular access strategies [4].

Patients with extensive comorbidities, including poorly controlled diabetes, severe hypertension, hyperlipidemia, coronary artery disease, and advanced chronic kidney disease (CKD), have higher rates of stroke and complications [5,6]. In particular, CKD often raises concerns about contrast-induced nephropathy. However, in a severe acute ischemic stroke, mechanical thrombectomy may still be warranted, given the life- or function-threatening nature of occlusion [7].

Here, we present a case of a 56-year-old woman with a history of multiple risk factors who sustained an acute occlusion of the anterior cerebral artery (ACA), uncovered to be supplied in part by a PTA arising from the right ICA. We detail the diagnostic process, including imaging and angiographic findings, the mechanical thrombectomy procedure, and the patient’s subsequent course and rehabilitation.

## Case presentation

Patient characteristics and presentation

A 56-year-old woman employed as a receptionist arrived at our Emergency Department (ED) after experiencing transient episodes of vomiting and imbalance, followed by loss of consciousness. She was discovered on the ground by her son with left-sided weakness, slurred speech, and gaze deviation, and she had reduced sensorium upon arrival. Her last known normal was documented as 4 hours and 48 minutes prior.

Her past medical history is significant for advanced chronic kidney disease (stage five), hypertension, dyslipidemia, type II diabetes mellitus, coronary artery disease, and heart failure. On examination, her blood pressure was severely elevated at 190/139 millimeters of mercury (mmHg). She was drowsy but arousable, with a Glasgow Coma Scale (GCS) score of 14 out of 15. Neurological evaluation revealed severe left-sided weakness, left facial droop, gaze deviation, intact visual fields, and sensory neglect. The National Institutes of Health Stroke Scale (NIHSS) score was 12. Her serum glucose level was 11.8 millimoles per liter.

Diagnostic criteria

The patient exhibited sudden neurological deficits localizing to the right hemisphere (left-sided hemiparesis and facial weakness), with onset within a timeframe (~4 hours), potentially allowing endovascular intervention.

Imaging findings

Initial non-contrast head computed tomography (CT) was performed 16 minutes after the patient’s arrival and demonstrated right middle cerebral artery (MCA) hyperdense signs, chronic microvascular ischemic changes, and an old hypodensity in the right cerebellum suggesting a previous infarct. Given her clinical presentation, which was concerning for a large vessel occlusion, a contrast-enhanced study was deemed necessary to assess intracranial vessels, as the potential benefits outweighed the associated risks.

**Figure 1:**
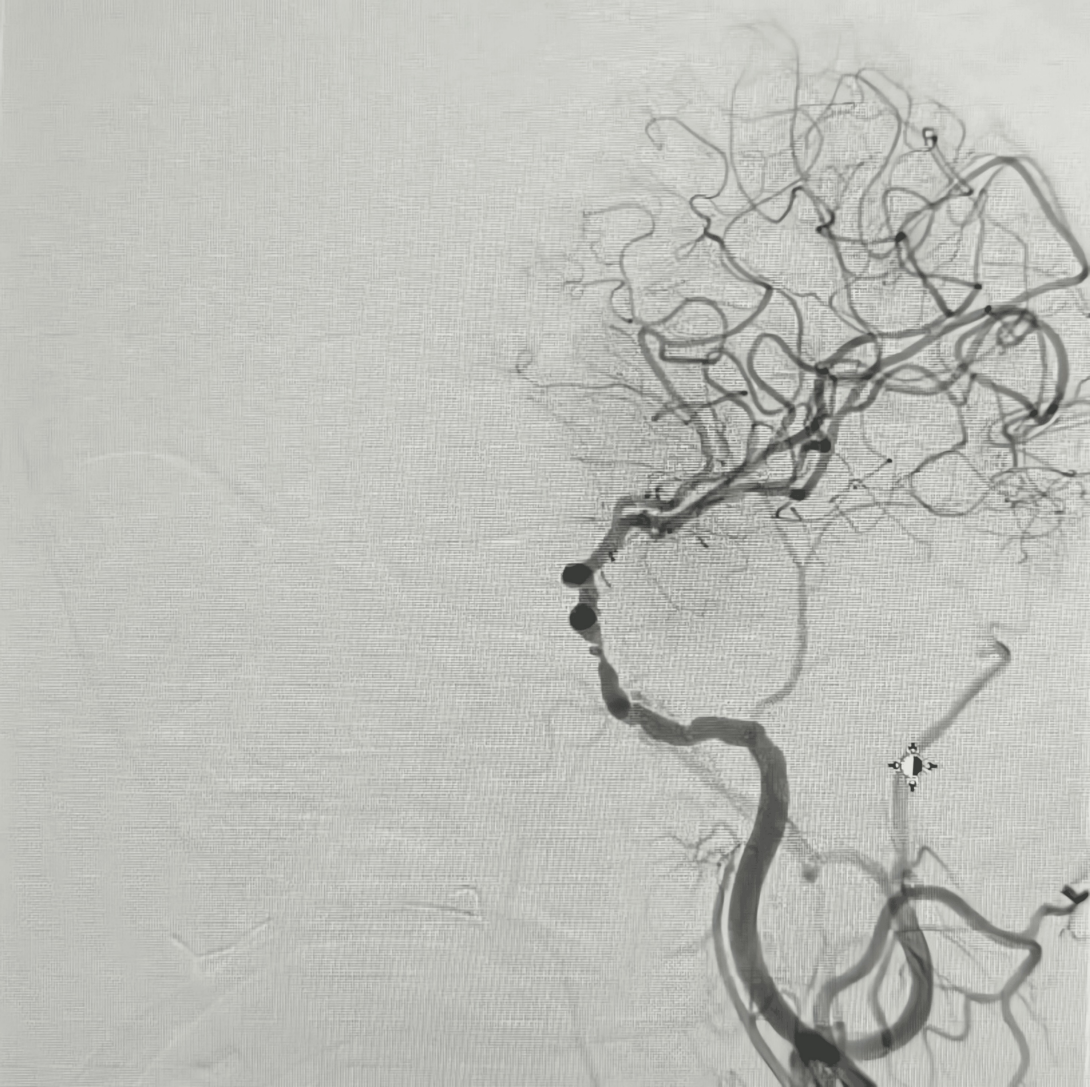
Digital subtraction angiography of the left internal carotid artery demonstrating severe intracranial atherosclerotic disease

Computed tomography angiography (CTA) was then performed 1 hour and 20 minutes later, after initial difficulty due to the patient’s uncooperative state and inability to remain still. It revealed a severely diminutive proximal segment of the basilar artery and corresponding vertebral arteries, raising concerns about high-grade stenosis and vertebrobasilar insufficiency. There was also evidence of severe stenosis in the right A2 segment of the anterior cerebral artery, along with significant atherosclerotic involvement of both distal internal carotid arteries. A CT perfusion scan was attempted but yielded poor-quality images due to patient motion and clinical instability. Although a persistent trigeminal artery (PTA) might occasionally be visualized on high-quality CTA, in this case, the suboptimal scan and pronounced atherosclerosis made detection difficult. Thus, the PTA was definitively appreciated only on digital subtraction angiography (DSA). Figure 1 illustrates the DSA findings obtained via a left internal carotid artery injection, demonstrating severe intracranial atherosclerotic disease.

Medical decision

Because of concerns regarding basilar artery insufficiency, we proceeded with an urgent DSA to allow for real-time therapeutic intervention if indicated. The arterial puncture was performed at 3 hours and 39 minutes after the patient’s arrival in the Emergency Room (ER). She was intubated and received general anesthesia for the procedure. The DSA revealed an occlusion of the right A2 segment of the anterior cerebral artery (ACA). Thrombectomy with aspiration was attempted and achieved thrombolysis in cerebral infarction (TICI) grade 2b recanalization. Although TICI scoring is most commonly discussed in the context of large vessel occlusions, it remains a widely used qualitative measure of reperfusion in endovascular procedures and can be applied to medium vessel occlusions such as the distal ACA. A persistent trigeminal artery was also identified.

Subsequent magnetic resonance imaging (MRI) of the brain confirmed an ischemic stroke in the right ACA territory. The patient was admitted to the intensive care unit (ICU) for post-thrombectomy care and aggressive medical management of intracranial atherosclerosis. An echocardiogram (commonly referred to as an “echo”) showed an ejection fraction (EF) of 40%, moderate left atrial (LA) enlargement, global hypokinesia, grade II diastolic dysfunction, and calcified aortic leaflets.

**Figure 2:**
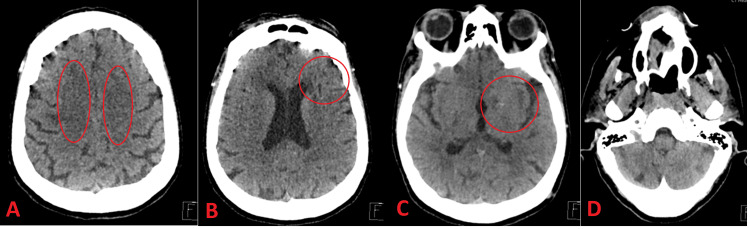
Noncontrast CT head demonstrating extensive chronic microvascular changes and an old infarction in the right cerebellum The CT scan shows bilateral periventricular white matter ischemic changes consistent with small vessel disease. A: Bilateral periventricular low‐attenuation areas consistent with chronic small‐vessel ischemic changes. B: Ongoing white matter changes, again suggestive of microvascular ischemia and more evident in the right frontal region. C: Extension of ischemic changes in the deep white matter; could reflect chronic lacunar infarcts or gliosis. D: Right cerebellar volume loss and encephalomalacia consistent with an old infarction.

**Figure 3:**
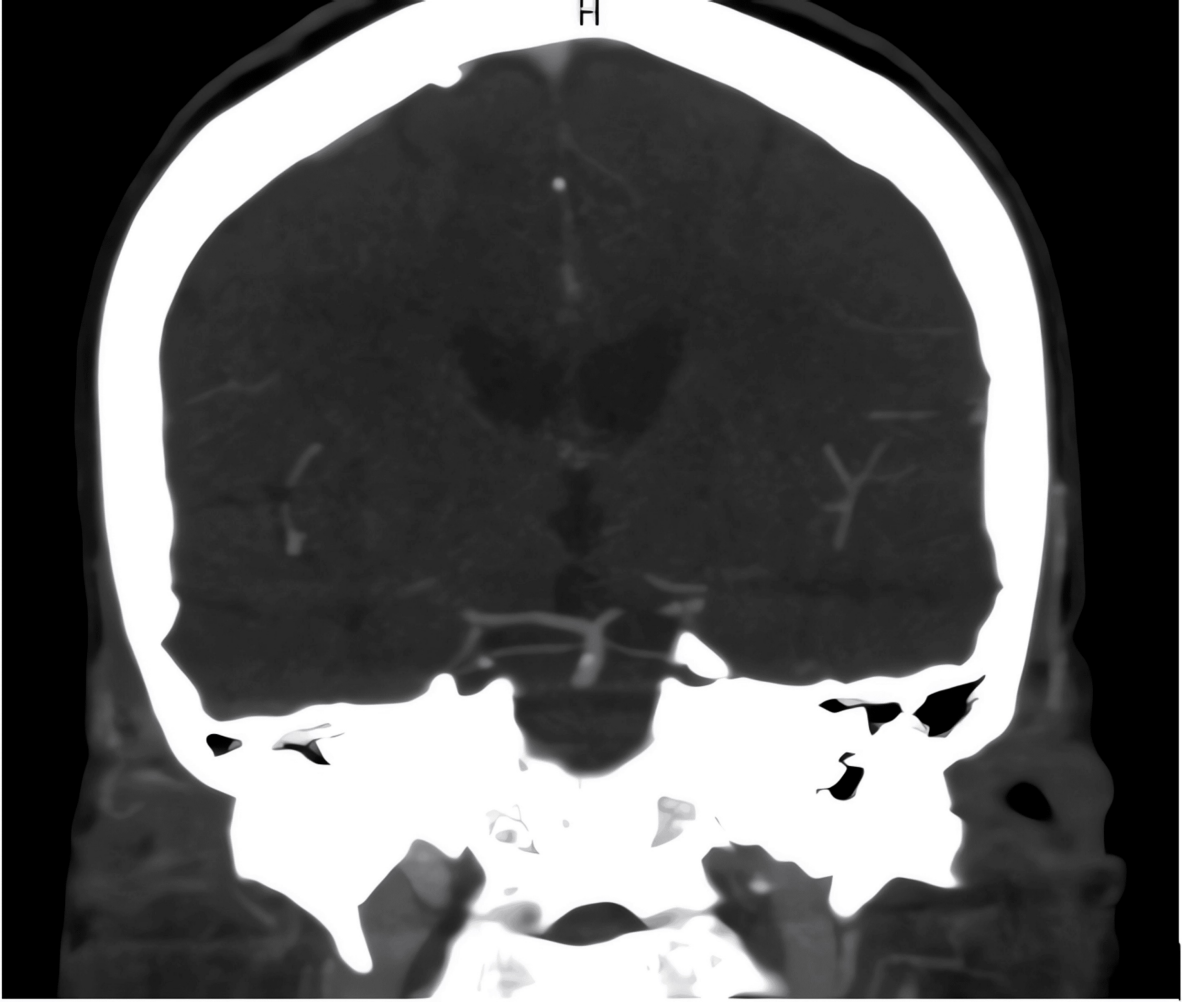
CT angiography demonstrating a proximal basilar artery cut-off, suggestive of an occlusion or high-grade stenosis.

During her admission, the patient required renal replacement therapy (dialysis), which continued upon discharge. Her modified Rankin Scale (mRS) score at discharge was 4. Figure 2 and Figure 3 show key sections of the patient’s initial neuroimaging, encompassing both noncontrast head computed tomography (CT) and subsequent CT angiography findings.

**Figure 4:**
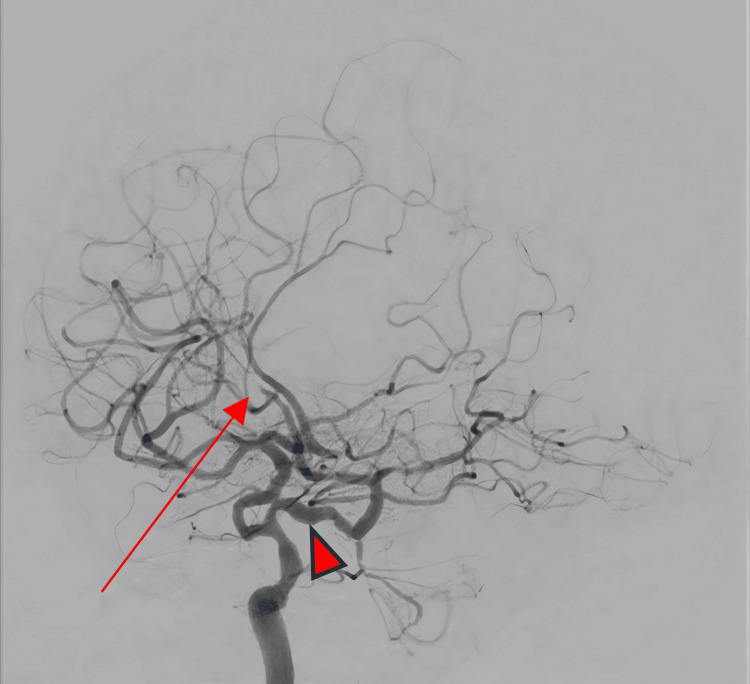
Oblique digital subtraction angiogram (right ICA injection) demonstrating the left ACA occlusion and a persistent trigeminal artery Digital Subtraction Angiogram obtained via a right internal carotid injection. Arrow: abrupt cutoff in the left A2 segment, indicating occlusion of the left anterior cerebral artery. Arrowhead: the persistent trigeminal artery branching posteriorly from the cavernous segment of the right ICA. Note the partial supply of the posterior circulation through this embryonic connection. ICA: internal carotid artery; ACA: anterior cerebral artery

Figure 4 presents an oblique digital subtraction angiogram obtained through the right internal carotid artery injection, clearly demonstrating the lack of contrast flow and consequent occlusion affecting the left anterior cerebral artery.

**Figure 5:**
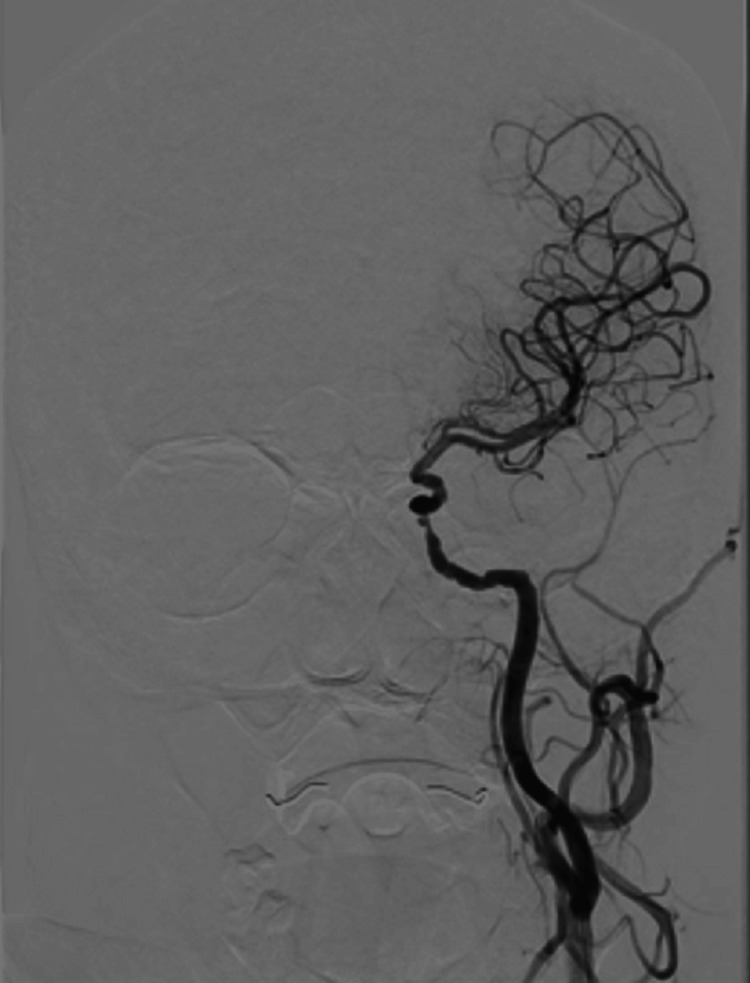
Digital subtraction angiography showing severe intracranial atherosclerotic disease of the left internal carotid artery

**Figure 6:**
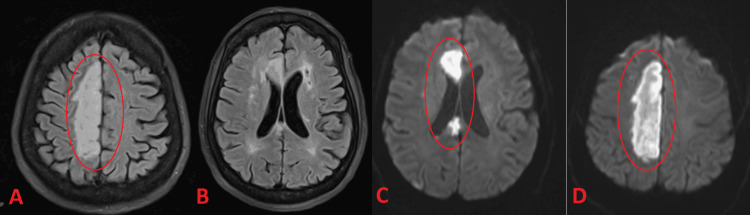
MRI confirming acute bilateral anterior cerebral artery (ACA) infarction A and B (FLAIR): A hyperintense signal is observed along the medial (parasagittal) surfaces of both frontal lobes, indicating acute infarcts in the ACA territory. Mild periventricular hyperintensities reflect chronic small‐vessel ischemic changes. C and D (DWI): A bright (restricted) signal in the same medial frontal regions confirms acute infarction bilaterally; the small‐vessel changes remain non‐restricted and chronic. FLAIR: fluid-attenuated inversion recovery; DWI: diffusion-weighted imaging

Figures 5-6 highlight the complementary findings of angiographic and MRI imaging in a patient with large-vessel disease. Figure 5 demonstrates severe intracranial atherosclerotic disease of the left internal carotid artery on digital subtraction angiography, while Figure 6 confirms an acute right anterior cerebral artery stroke on MRI.

Laboratory findings

Table 1 shows the key laboratory results.

**Table 1:** Laboratory findings Abnormal values are marked with an asterisk. -LDL cholesterol: <2.6 mmol/L is often considered optimal, 2.6–3.3 mmol/L near optimal/above optimal, 3.4–4.1 mmol/L borderline high, 4.1–4.9 mmol/L high, and >4.9 mmol/L very high. -HbA1c: <5.7% is typically regarded as normal, 5.7–6.4% indicates prediabetes, and ≥6.5% suggests diabetes mellitus. LDL: low-density lipoprotein; HbA1c: glycated hemoglobin

Test	Result	Reference Range
Hemoglobin	115 g/L	(120–160 g/L)*
Hematocrit	0.34 L/L	(0.37–0.47 L/L)*
Mean Corpuscular Volume (MCV)	70.8 fL	(80–100 fL)*
Creatinine	632 µmol/L	(53–106 µmol/L)*
Urea	28.3 mmol/L	(2.1–7.1 mmol/L)*
Estimated Glomerular Filtration Rate (eGFR)	~6 mL/min	(>90 mL/min)*
Glucose	11.8 mmol/L	(3.3–5.8 mmol/L)*
Troponin T	350.0 ng/L	(<14 ng/L)*
Activated Partial Thromboplastin Time (aPTT)	24.1 s	(25–35 s)
Bicarbonate	18 mmol/L	(22–29 mmol/L)*
Lactate	2.3 mmol/L	(0.5–2.2 mmol/L)*
Low-Density Lipoprotein (LDL) Cholesterol	0.72 mmol/L	(<2.6 mmol/L)**
Hemoglobin A1c (HbA1c)	7.0%	(<5.7%)**

Notable abnormalities included advanced renal impairment (eGFR ~6 mL/min), hyperglycemia, mild anemia, and elevated troponin T (possibly reflecting demand ischemia).

Risk stratification and considerations management

Given her long-standing diabetes, severe hypertension, and advanced CKD, the patient was at high risk for both ischemic events and peri-procedural complications. Still, the potential benefit of mechanical thrombectomy outweighed the risk of further renal compromise, particularly as intravenous (IV) thrombolysis was contraindicated due to time and renal considerations.

Pharmacological management

Blood pressure (BP) was initially controlled with intravenous (IV) nicardipine infusion, reducing it from 190/139 mmHg to below 185/110 mmHg before endovascular therapy. Antiplatelet therapy was administered with aspirin 300 mg and clopidogrel 300 mg loading doses. Although the patient arrived close to the 4.5-hour thrombolytic window, her documented last known well was at 4 hours and 48 minutes, which exceeded the recommended limit for IV thrombolysis. In addition, her advanced CKD raised further concerns regarding the safety of IV thrombolysis. Therefore, IV thrombolysis was not administered. Supportive care included judicious IV fluids to maintain perfusion without aggravating fluid overload and continuous cardiac monitoring due to elevated troponin and coronary artery disease.

Interventional management (mechanical thrombectomy)

After CTA confirmed anterior cerebral artery occlusion (ACAO), an urgent mechanical thrombectomy was performed. DSA revealed the persistent trigeminal artery (PTA) arising from the right ICA and severe intracranial atherosclerosis affecting the anterior cerebral artery origin. Multiple passes with a stent retriever device achieved successful recanalization. Contrast administration was minimized but deemed necessary given the life-threatening nature of large-vessel occlusion.

Rehabilitation and post-procedural care

Following the procedure, the patient was admitted to the intensive care unit (ICU) for close hemodynamic and neurologic monitoring. A repeat non-contrast head CT demonstrated no hemorrhage and partial resolution of early ischemic changes, indicating reperfusion. Despite restored arterial patency, she continued to exhibit significant left-sided weakness and dysarthria, necessitating intensive neurorehabilitation with physical, occupational, and speech therapy. Long-term secondary prevention measures included stricter glycemic control, aggressive BP management, statin therapy, and dual antiplatelet agents initially (transitioning to single antiplatelet later, following standard protocols).

## Discussion

ACAO can lead to severe neurological deficits if not promptly recanalized [1,2]. While mechanical thrombectomy has been widely embraced for large vessel occlusions in the anterior circulation, anatomic variations like a persistent trigeminal artery (PTA) can alter expected hemodynamic patterns and challenge conventional intervention strategies [3,4].

Our patient’s multiple comorbidities, particularly severe hypertension and advanced diabetes, amplified her risk for cerebrovascular events and limited collateral circulation [5,6]. Advanced chronic kidney disease (CKD) raised concerns about contrast-induced nephropathy, but emergent mechanical thrombectomy is often justified in life- or function-threatening stroke situations [7].

A persistent trigeminal artery (PTA) is an embryologic remnant that can connect the internal carotid artery (ICA) to other arteries, sometimes the vertebrobasilar system, and in rare configurations, variants of the anterior circulation [3,4]. In the setting of an ACAO, the PTA can provide partial collateral flow but may also complicate the procedural approach. Recognition of this variant on angiography is critical for planning access, device selection, and stent retriever positioning.

Despite successful recanalization, our patient’s neurological recovery was limited, reflecting: delayed reperfusion (even short delays in large vessel occlusions can drastically affect outcomes [8], severe intracranial atherosclerosis (compromised collateral routes and a heightened risk of re-occlusion [9]), and multiple comorbidities (long-standing diabetes and hypertension) exacerbate small-vessel disease, increase tissue damage, and hamper neuroplasticity, limiting rehabilitation potential [5,6].

Limitations

A persistent trigeminal artery (PTA) in the setting of anterior circulation occlusion is rare, making standardized road map protocols difficult to establish. Suboptimal initial imaging, compounded by motion artifacts and the patient’s clinical instability, delayed definitive diagnosis. Given its rarity, a practical road map might include: (1) early recognition on initial vascular imaging, (2) prompt multidisciplinary discussion among neurology, neuroradiology, and neurosurgery, (3) careful assessment of hemodynamic significance and collateral flow, (4) individualized risk-benefit evaluation in patients with complex comorbidities (e.g., advanced CKD), and (5) strategic choice of endovascular devices and techniques to accommodate unusual arterial routes. Furthermore, as a single-case design, these findings are not generalizable; more extensive studies or registries are needed to clarify best practices in such complex arterial variants.

Clinical relevance

This case underscores the importance of several factors. Rapid recognition through urgent neuroimaging and vascular studies remains paramount. An adaptive endovascular strategy, bolstered by an understanding of atypical vascular connections, such as a PTA, is essential for safe and effective recanalization. Aggressive risk factor control, optimally managing hypertension, diabetes, and chronic kidney disease (CKD), may reduce initial stroke risk and improve recovery. Finally, comprehensive rehabilitation, with early and intensive therapy, is crucial post-thrombectomy, particularly for patients with substantial comorbidities and severe deficits.

Recommendations for future practice

High-resolution vessel imaging, including advanced vessel-wall imaging or magnetic resonance (MR) angiography, can clarify arterial anatomy and collateral pathways in patients with suspected vascular variants. Robust registry data, particularly from multi-center efforts documenting unusual vascular connections in anterior circulation large vessel occlusions, can guide standardized approaches. Precision in risk factor management, such as strict glycemic and blood pressure (BP) control, could lower both initial stroke risk and recurrence in high-risk groups. Lastly, customized rehabilitation protocols targeting advanced comorbidities may optimize functional outcomes.

## Conclusions

Anterior cerebral artery occlusion in the presence of a persistent trigeminal artery and multiple significant comorbidities presents a formidable treatment challenge. In this case, the recognition of this rare anatomic variant, combined with extensive comorbidities, such as advanced kidney disease, was critical to planning a safe and effective endovascular approach. Although mechanical thrombectomy achieved vessel recanalization, the patient’s neurological recovery remained limited, emphasizing that timely intervention, robust collateral circulation, and aggressive risk factor management are equally vital to optimize functional outcomes. Mechanical thrombectomy, while technically successful, may not always guarantee full neurological recovery, particularly if presentation is delayed and underlying atherosclerosis is extensive. Nonetheless, rapid recognition, collaborative multidisciplinary management, and prompt intervention remain the keystones of improving functional outcomes in anterior circulation stroke complicated by rare anatomic variants and systemic disease. This case illustrates the importance of comprehensive patient evaluation, including the assessment of unusual vascular anomalies, and underscores the need for ongoing research to better understand the interplay between advanced comorbidities, vascular variants, and stroke outcomes.
